# Supplementary material for: Microscope-Cockpit: Python-based bespoke microscopy for bio-medical science
Source: Wellcome Open Res. 2022 Jan 17;6:76. Originally published 2021 Apr 8. [Version 2] doi: 10.12688/wellcomeopenres.16610.2 (PMC10240544; doi:10.12688/wellcomeopenres.16610.2)
Supplement: Peer review history

# Peer review: Reviewer response for version 2

**DOI:** 10.21956/wellcomeopenres.19384.r58035

**Published:** 2023-06-21

**Authors:** Xavier Casas Moreno

**Affiliations:** 1 KTH Royal Institute of Technology, Stockholm, Sweden

The article presents Microscope-Cockpit, a software solution for microscope control that uses a software model for controlling multiple devices and implements a graphical user interface (GUI) which is user friendly for biologists. The authors present several use-cases that are promising and position Cockpit as a state-of-the-art tool. I have some comments that I would like the authors to address, after which the article should be ready for indexing.

 The authors compare Microscope-Cockpit with different state-of-the-art alternatives, such as LabView, uManager and custom code. However, in the recent years (2021-2023) there have been different initiatives to provide similar solutions in Python. How does Microscope-Cockpit relate to these efforts, and how do they complement each other? Some examples are ImSwitch, PycroManager, PYME, miEye, etc. In my opinion mentioning these will strengthen the current efforts in Python for microscope control.

 The authors use the dataformat ".dv". I miss a comparison between this and the state-of-the-art in microscopy research (tiff, hdf5 and ome-zarr). They are explained and compared here: https://www.nature.com/articles/s41592-021-01326-w. In case someone wants to provide support for these other formats, how difficult would it be to do so in Microscope-Cockpit?

 It is mentioned that Cockpit does not require a direct connection since it uses Pyro. Is Pyro always needed or is it an option when controlling remote objects? How does it work when the devices are in the local machine? And how are both approaches compared in terms of latency?

 The authors explain briefly the software model of Microscope-Cockpit, using devices and handlers, and the interfaces. I think it would be helpful to have a figure with a diagram of the software model, to see how the different layers relate to each other. Also, is the software model implementing a software design pattern?

 Thank you in advance.

Are the conclusions about the tool and its performance adequately supported by the findings presented in the article?

Yes

Is the rationale for developing the new software tool clearly explained?

Partly

Is the description of the software tool technically sound?

Yes

Are sufficient details of the code, methods and analysis (if applicable) provided to allow replication of the software development and its use by others?

Partly

Is sufficient information provided to allow interpretation of the expected output datasets and any results generated using the tool?

Yes

Reviewer Expertise:

Super-resolution microscopy, including software for microscope control.

I confirm that I have read this submission and believe that I have an appropriate level of expertise to confirm that it is of an acceptable scientific standard, however I have significant reservations, as outlined above.

# Peer review: Reviewer response for version 2

**DOI:** 10.21956/wellcomeopenres.19384.r58034

**Published:** 2023-06-02

**Authors:** Adam Wollman

**Affiliations:** 1 Newcastle University, Newcastle upon Tyne, England, UK

Microscope-Cockpit is an open source python software package for controlling bespoke microscopes. Although there is existing software for this (e.g. micro-manager), more competition in this space can only be a good thing for the microscopy community. Cockpit also has a number of advantages over other open source microscopy software. It is entirely based in python, making it natively compatible with lots of existing image software in python and easily built upon by the large python community. It has a touch screen interface and it is explicitly designed around mosaic imaging of multiple connected fields of view. This latter feature also leads to its two small possible weak points. The choice of file format is understandable considering mosaic imaging but it would have been nice to see native support for other image formats e.g. ome.tif . The software also appears to require a motorised x,y,z stage which again is understandable for mosaic imaging but not a requirement for lots of other types of microscope. Also, could the authors clarify, does this statement, ‘In addition, experiments require a device that can be programmed as source of hardware triggers, supplying digital signals and, optionally, analogue voltages.’ mean Cockpit will also only work with a control board for hardware triggers? Or will it work to some degree without?

 Otherwise Cockpit will be an extremely useful tool for the microscopy community going forward.

Are the conclusions about the tool and its performance adequately supported by the findings presented in the article?

Yes

Is the rationale for developing the new software tool clearly explained?

Yes

Is the description of the software tool technically sound?

Yes

Are sufficient details of the code, methods and analysis (if applicable) provided to allow replication of the software development and its use by others?

Yes

Is sufficient information provided to allow interpretation of the expected output datasets and any results generated using the tool?

Yes

Reviewer Expertise:

Microscopy development, image analysis software

I confirm that I have read this submission and believe that I have an appropriate level of expertise to confirm that it is of an acceptable scientific standard.

# Peer review: Reviewer response for version 2

**DOI:** 10.21956/wellcomeopenres.19384.r58030

**Published:** 2023-06-01

**Authors:** Marijonas Tutkus

**Affiliations:** 1 Center for Physical Sciences and Technology, Vilnius, Lithuania

This paper describes “Microscope-Cockpit” (Cockpit), which is adaptable open-source user-friendly Python-based Graphical User Interface (GUI). For hardware control it is using “Python-Microscope” (Microscope), and it includes coordinated control of hardware devices. It was verified using several bespoke microscopes, including a simple widefield system and a complex system with adaptive optics and structured illumination. It is written in the text that this system allows for using several cameras, but I did not see any actual demonstration of simultaneous operation with several cameras. 

 This Cockpit GUI allows for control of bespoke microscope systems, navigation of the entire slide landscape, and automated acquisition without the use of eyepieces. One of the functions that I miss a lot in this work is any sample auto-focusing pipeline. Sample's Z drift correction using IR laser back reflection or similar approaches became very popular and important for many experiments.

 A key strength of Cockpit is its use of Python, which means that any microscope built with Cockpit is ready for future customisation by simply adding new libraries, for example machine learning algorithms to enable automated microscopy decision making while imaging. However, I would like to make a note that making a universal software platform for any bespoke microscopy system is a challenging task, and probably each new case will require readjustments of the Cockpit.

 In this paper several software packages are compared to the Cockpit (such as LabVIEW, Matlab, µManager). It is important to mention that there are many more open-source alternatives that µManager that are able to do either hardware control or data analysis or both. Most of them are listed here. Recently newly developed open-source Python-based systems, such as the Sync-Scope,1 and miEye2 appeared. They both allow for similar functionality as the Cockpit. 

 Other specific comments.

1. It is written on page 3 “Many bespoke microscope systems that lack eyepieces, such as systems built by physical scientists in collaboration with biologists, make sample navigation particularly challenging.”. To be precise, it all depends on what is the sample. If one is looking into diffraction limited spots that are dim, looking through eyepieces would not help to navigate around the sample.
2. On page 4 it is written that “the principal design goals for the Cockpit interface were to” …. “Hide from the user the complexity required to control an advanced microscope”. I think it should be intuitive, not necessarily hidden.
3. On page 7: “Experimental images are saved into files utilising the ‘.dv’ file format,”. I would like to see explanation why authors decided not to use tiff or zarr formats.
4. On page 7: “the mosaic window enables navigation of the sample relative to an overview formed of tiled images;”. Does this GUI window allows for any data analysis on site? e.g. applying different LUT, adjusting intensity scale, etc.
5. On page 7: “Cockpit requires Python 3.5 or later”. Different Python versions often create compatibility problems. Did you check functionality of the Cockpit in different versions of Python?
6. On page 10: “The system has 4× and 63× air objectives. Although changing the objective involves physically removing the objective and its mount, this is easily achieved due to the spring-loaded kinematic mounting system.”. Doesn’t that mean that one has to remove the sample when changing objectives? In such a case, returning back to the original positions would be challenging.
7. On page 10: “If a live sample is used the system can be used for time lapse imaging.”. Probably any dynamic sample can be used for time lapse imaging regardless it is living cells or just molecules in vitro.
8. On page 11: “We have developed a Python-based GUI for controlling bespoke microscopes which can map the entire slide or dish, allowing a real time exploration of the entire specimen landscape.”.  I suggest using "interactive exploration".
9. On page 12: “This has the additional benefit that connected hardware may be physically located on another computer, increasing scalability and allowing devices requiring incompatible hardware or software to still be seamlessly integrated.”. In such case latency might become an issue. Please, provide explanation on this matter.
10. On page 12: “We  have ranked features of the control software options with a traffic light colour scheme with green being best and red least good. The table includes the approaches mentioned above along with Matlab, a common alternative to LabVIEW, but a more conventional programming package with a range of extensions and with a large support base, and MetaMorph (https://www. moleculardevices.com/products/cellular-imaging-systems/acquisition- and-analysis-software/metamorph-microscopy) / SlideBook (https://www.intelligent-imaging.com/slidebook) included as representatives of commercial generalised microscope control packages.”. I think, Igor Pro is also used relatively widely in the community to control microscope's hardware and perform data analysis.

Are the conclusions about the tool and its performance adequately supported by the findings presented in the article?

Yes

Is the rationale for developing the new software tool clearly explained?

Yes

Is the description of the software tool technically sound?

Yes

Are sufficient details of the code, methods and analysis (if applicable) provided to allow replication of the software development and its use by others?

Yes

Is sufficient information provided to allow interpretation of the expected output datasets and any results generated using the tool?

Yes

Reviewer Expertise:

single-molecule biophysics, super-resolution microscopy, image analysis, open-source microscopy.

I confirm that I have read this submission and believe that I have an appropriate level of expertise to confirm that it is of an acceptable scientific standard, however I have significant reservations, as outlined above.

## References

[1] : An open-source microscopy framework for simultaneous control of image acquisition, reconstruction, and analysis. *HardwareX* .2023;13: 10.1016/j.ohx.2023.e00400 e00400 10.1016/j.ohx.2023.e00400 36824447PMC9941414

[2] : The miEye: Bench-top super-resolution microscope with cost-effective equipment. *HardwareX* .2022;12: 10.1016/j.ohx.2022.e00368 e00368 10.1016/j.ohx.2022.e00368 36248253PMC9556790

# Peer review: Reviewer response for version 2

**DOI:** 10.21956/wellcomeopenres.19384.r58037

**Published:** 2023-06-01

**Authors:** Joe Knapper

**Affiliations:** 1 Physics and Astronomy, University of Glasgow, Glasgow, Scotland, UK

I'd like to thank the authors for sharing this work, based around making the control of microscopy systems more intuitive for the user based on a GUI for Python-Microscope. I agree with the suggestions of the first round of reviews, and agree with the authors that these changes improved the article. While I recommend the article and tool are ready for publication, I would like to raise the following questions.

 The inclusion of Table 1 gives more context for Microscope-Cockpit, but I hope the authors can share how it was assessed? As a subjective ranking, is it based on the feedback of multiple users and developers, public forums, etc?

 Can the authors expand on the ease of use of exporting the '.dv' format? I note they list it is supported in ImageJ, OMERO, Matlab and Chromagnon, but as this work is Python-based, can the authors suggest a (preferably native) Python-compatible solution? omero-py, pyimagej, or ideally something only requiring a pypi installation? The first sentence of "Output image files" is also hard to follow - does a single '.dv' file contain multiple images, wavelengths, z-slices and time points?

 The GPU-based mosaic for multiple large area scans appears to be an important feature of this work, and very useful for potential users. Can the authors clarify if this is positioning and blending images together based on areas of overlap, or simply placing images directly based on the stage position?

 Finally, a note to the authors that currently it appears that Python >=3.10 is incompatible, based on wxPython depending on attrdict. Whether this is addressed in the paper or the program is up to the authors.

Are the conclusions about the tool and its performance adequately supported by the findings presented in the article?

Yes

Is the rationale for developing the new software tool clearly explained?

Yes

Is the description of the software tool technically sound?

Yes

Are sufficient details of the code, methods and analysis (if applicable) provided to allow replication of the software development and its use by others?

Yes

Is sufficient information provided to allow interpretation of the expected output datasets and any results generated using the tool?

Yes

Reviewer Expertise:

Python, microscope control, image tiling

I confirm that I have read this submission and believe that I have an appropriate level of expertise to confirm that it is of an acceptable scientific standard.

# Peer review: Reviewer response for version 1

**DOI:** 10.21956/wellcomeopenres.18310.r45668

**Published:** 2021-09-29

**Authors:** Jason R Swedlow, Emil Rozbicki, William Moore, Sébastien Besson

**Affiliations:** 1 Centre for Gene Regulation and Expression, School of Life Sciences, University of Dundee, Dundee, UK; 2 Glencoe Software, Inc, Seattle, WA, USA; 3 OME, University of Dundee, Dundee, UK

This paper introduces Microscope-Cockpit, a graphical user interface software that has been built on top of the historical software used to control the OMX microscope. It aims to provide a clean and user friendly interface allowing to create complex experimental set-up. Several use cases are included in the article to support the capacity of the software to adapt to different experimental set-ups. Overall the tool described in this article is scientifically valid but we would like to suggest some revisions detailed below.

 The Implementation section details the Python requirements of Microscope-Cockpit. Amongst those packages, the Python-microscope library appears as one of the most critical dependencies since it contains all the logic and support for handling and controlling microscopy devices. As part of this review, it came to our attention that this library has been recently published ( https://doi.org/10.1242/jcs.258955)​​ ^1^ including a supplementary figure Fig S1 which cross-references Fig 1 of this paper. The article should thus be amended to cross-reference the Python-microscope publication wherever appropriate.

 The article makes no mention of the file format written by Microscope-Cockpit. The Python-microscope paper does not include any detail about the choice of data output for end-users either. From our minimal testing of the application, the only option available to the user is to write data as “DV files (.dv)”, a proprietary file format. Is this the only choice available to the user? If so, this feels like a limitation of the current implementation which contradicts the claim for the open and universal mission of Cockpit. We suggest a paragraph should be added to the Implementation section that discusses the Data Generation/Output including a description of the file format as well as the tooling available for end-users of the acquisition system to read and analyze imaging data generated by Cockpit.

 In the section discussing Cockpit in relationship Micro-Manager, a proposed key strength of Cockpit is its Python implementation allowing the “strict separation of the user interface from the hardware control components” while “the majority of [Micro-Manager] interface must be written in Java; however the system also needs a C/C++ layer to interface the Java code to C/C++ based system libraries”. This argument omits Pycro-Manager, a Python bridge for Micro-Manager, published earlier this year ( https://doi.org/10.1038/s41592-021-01087-6) ^2^ and mentioned in the aforementioned Python-microscope publication. The section mentioned above should be amended to cite this paper and include Pycro-Manager in the comparison with Cockpit.

 Much is made of the ability to use Python for custom modifications and the integration of third-party libraries. The text mentions that “the microscope control can easily be modified to make use of the extensive machine learning algorithms available in easy to integrate libraries” but this sentence rather suggests the extensibility is a property of the underlying python-microscope library rather than the Cockpit interface. The Cockpit reference documentation ( https://www.micron.ox.ac.uk/software/cockpit/) primarily describes the installation and the configuration of the software. To support the extensibility claim, we would suggest either to introduce a public example of Python extension to the default Cockpit UI and/or an amendment to the online documentation that would demonstrate this extension which could be linked from the article.

 Together with python-microscope paper, this paper introduces a new open-source solution for microscope control and imaging acquisition. Both in the introduction and in the discussion, Cockpit is compared to several equivalent commercial and open-source solutions, including the well-established Micro-Manager. This comparison is important and particularly useful for end users and imaging facility managers who need to make informed decisions e.g. when investing in new equipment or technologies. We suggest to make the outcome of this discussion more explicit and prominent e.g. under a form of a summary table that would include the different available ecosystems (individual hardware solutions, LabView, python-microscope/Microscope-Cockpit and Micro-Manager/Pycro-Manager) and compare their respective advantages and drawbacks.

Are the conclusions about the tool and its performance adequately supported by the findings presented in the article?

Yes

Is the rationale for developing the new software tool clearly explained?

Partly

Is the description of the software tool technically sound?

Yes

Are sufficient details of the code, methods and analysis (if applicable) provided to allow replication of the software development and its use by others?

Yes

Is sufficient information provided to allow interpretation of the expected output datasets and any results generated using the tool?

No

Reviewer Expertise:

bioimaging, image informatics, software engineering, data management

We confirm that we have read this submission and believe that we have an appropriate level of expertise to confirm that it is of an acceptable scientific standard, however we have significant reservations, as outlined above.

## References

[1] : Python-microscope: A new open source Python library for the control of microscopes. *J Cell Sci* .2021; 10.1242/jcs.258955 10.1242/jcs.258955 34448002PMC8520730

[2] : Pycro-Manager: open-source software for customized and reproducible microscope control. *Nature Methods* .2021;18(3) : 10.1038/s41592-021-01087-6 226-228 10.1038/s41592-021-01087-6 PMC853217633674797

# Response

**Published:** 2021-12-14

**Authors:** Ian Dobbie

**Affiliations:** University of Oxford, UK

Response to review from authors. 

 Authors responses are in *italics*

 New text in the manuscript are in **bold**

 The Implementation section details the Python requirements of Microscope-Cockpit. Amongst those packages, the Python-microscope library appears as one of the most critical dependencies since it contains all the logic and support for handling and controlling microscopy devices. As part of this review, it came to our attention that this library has been recently published ( https://doi.org/10.1242/jcs.258955)​​ ^1^ including a supplementary figure Fig S1 which cross-references Fig 1 of this paper. The article should thus be amended to cross-reference the Python-microscope publication wherever appropriate.

*We thank the reviewers for pointing this out. The Python-Microscope publication has been accepted at JCS since we submitted the article. We have revised the manuscript to reference the published article rather than the biorXiv preprint.*

 The article makes no mention of the file format written by Microscope-Cockpit. The Python-microscope paper does not include any detail about the choice of data output for end-users either. From our minimal testing of the application, the only option available to the user is to write data as “DV files (.dv)”, a proprietary file format. Is this the only choice available to the user? If so, this feels like a limitation of the current implementation which contradicts the claim for the open and universal mission of Cockpit. We suggest a paragraph should be added to the Implementation section that discusses the Data Generation/Output including a description of the file format as well as the tooling available for end-users of the acquisition system to read and analyze imaging data generated by Cockpit.

*We thank the reviewers for raising this point. While it is correct that Microscope-Cockpit only saves images in the ‘.dv’ file format, it is a slight variant of the open MRC file format commonly used in the electron microscopy community. The MRC format is extensively documented at https://www.ccpem.ac.uk/mrc_format/mrc2014.php. The sight variations to the encoded metadata reflect differences between the relevant data in optical and electron microscopy. Crucially, there is considerable support within open source software that supports ‘.dv’ files, including Bio-Formats for importing data into ImageJ, OMERO and Matlab. Therefore, users can easily convert to other formats and use popular programs to analyse the data output by Microscope-Cockpit based microscopes.  We have revised the manuscript to address this point and changed the software package documentation to include an explanation of this as well as information about the software that supports the ‘.dv’ format, and explicit definition of what metadata we save in the files. We have added a section on output image files which explicitly mentions these points:*

**Output image files**

**Experimental images are saved into files utilising the ‘.dv’ file format, typically multiple wavelengths, Z-slices and time points into a single file. Images from the mosaic window and single snaps can also be saved into this format, with the mosaic saved files having an associated text file defining XYZ positions of each collected image. The ‘.dv’ format is an extension of the mrc file format, defined in detail in the MRC/CCP4 2014 file format specification. The CCP4 consortium of the EM community continue to support and extend this file format. This support includes file validators and a detailed specification, which is compatible with the files used here but not identical.**

**The optical microscopy specific metadata are covered in the documentation. Although relatively uncommon, the file format is supported by the Bio-Formats project allowing import of ‘.dv’ files, along with the associated metadata into software using this library including ImageJ, OMERO and Matlab. Additionally, the Chromagnon image alignment tool will read and write ‘.dv’ files and it is the native format for DeltaVision microscopes utilising the commercial package SoftWoRx.**

 In the section discussing Cockpit in relationship Micro-Manager, a proposed key strength of Cockpit is its Python implementation allowing the “strict separation of the user interface from the hardware control components” while “the majority of [Micro-Manager] interface must be written in Java; however the system also needs a C/C++ layer to interface the Java code to C/C++ based system libraries”. This argument omits Pycro-Manager, a Python bridge for Micro-Manager, published earlier this year ( https://doi.org/10.1038/s41592-021-01087-6) ^2^ and mentioned in the aforementioned Python-microscope publication. The section mentioned above should be amended to cite this paper and include Pycro-Manager in the comparison with Cockpit.

*We thank the reviewers for pointing out this paper which was again published since our original submission and we have now added discussion about Pycro-Manager to the paper. We have revised the manuscript by adding the following text to the discussion section:*

**It should be noted that Pycro-Manager [pinkard2021] has recently been developed and released to allow much closer integration between Python and uManager. Pycro-Manager seems likely to fill a similar position to Microscope-Cockpit, allowing direct Python based control of imaging experiments, integration with online analysis and a range of other functionality. As this is a recent development, we have not explored this package in any detail.**

 Much is made of the ability to use Python for custom modifications and the integration of third-party libraries. The text mentions that “the microscope control can easily be modified to make use of the extensive machine learning algorithms available in easy to integrate libraries” but this sentence rather suggests the extensibility is a property of the underlying python-microscope library rather than the Cockpit interface. The Cockpit reference documentation ( https://www.micron.ox.ac.uk/software/cockpit/) primarily describes the installation and the configuration of the software. To support the extensibility claim, we would suggest either to introduce a public example of Python extension to the default Cockpit UI and/or an amendment to the online documentation that would demonstrate this extension which could be linked from the article.

*The reviewers make a good point that would significantly strengthen the paper. We have written a short script that utilises some of the embedded functionality to find and mark cell nuclei in a typical DAPI stained sample. The code, along with a test image set, config files and detailed instructions on how to set it up are collected into a zenodo repository and available via a doi (10.5281/zenodo.5745648) link.  In fact, this simulated microscope approach is a very powerful approach to setting up and testing such scripts. We have also added the following text to the manuscript.*

**Example simple extension in Python**

**We include an example script which demonstrates how the functionality of cockpit can easily be extended in python. The script finds DAPI stained cell nuclei in images, utilising the mosaic functionality to scan large areas and the point marking features to record the centroids of the detected nuclei. The script utilises the OpenCV framework to detect large roughly circular objects in images. Images taken for the mosaic are also trapped by the code which Gaussian blurs to reduce noise, binarise and finally applies a Hough transform to find circular objects. The locations of these objects in the image are then transformed into stage coordinates and added to the marked point list. The Python code, a test image and detailed instructions on how to setup a simulated microscope to run this without any microscope hardware is detailed at https://doi.org/10.5281/zenodo.5745648.**

**For this simple example the parameters are fixed and tuned to the data set used, and select a subset of nuclei within a size range and with defined circularity. The current code also doesn’t reliably detect nuclei at the edges between images in the mosaic scan. However, it can easily detect a large number of cell positions to create a point list which could then be run through a multi-site experiment to collect data such as a 3D, multi-channel stack, or time lapse on a large number of cells semi-automatically.**

 Together with python-microscope paper, this paper introduces a new open-source solution for microscope control and imaging acquisition. Both in the introduction and in the discussion, Cockpit is compared to several equivalent commercial and open-source solutions, including the well-established Micro-Manager. This comparison is important and particularly useful for end users and imaging facility managers who need to make informed decisions e.g. when investing in new equipment or technologies. We suggest to make the outcome of this discussion more explicit and prominent e.g. under a form of a summary table that would include the different available ecosystems (individual hardware solutions, LabView, python-microscope/Microscope-Cockpit and Micro-Manager/Pycro-Manager) and compare their respective advantages and drawbacks.

*We thank the reviewers for this suggestion and we have added a table of feature comparison between a range of microscope control options as a traffic light colour coded table and the following text to the manuscript.*

**Table 1 shows a comparison between various features of the previously described microscope control software options. We have ranked features of the control software options with a traffic light colour scheme with green being best and red least good. The table includes the approaches mentioned above along with Matlab, a common alternative to LabVIEW, a more conventional programming package with a range of extensions and with a large support base, and MetaMorph/ SlideBook included as representatives of commercial generalised microscope control packages.**

**[New table included in revised version]**

**Table 1: A symbolic representation of the relative strengths of different microscope control approaches in a number of areas with a traffic light colour scheme, with red worst and green best. Separate control programs involve using a separate control program for each piece of hardware. In general, this software is free, however some components such as cameras might require a separate software purchase from the manufacturer. MetaMorph and SlideBook are examples of generalised commercial software designed for microscope control and provided by third parties, these are only two examples of a range of such packages.**

# Peer review: Reviewer response for version 1

**DOI:** 10.21956/wellcomeopenres.18310.r45664

**Published:** 2021-09-14

**Authors:** Kyle Harrington

**Affiliations:** 1 Max Delbrueck Center for Molecular Medicine, Berlin, Germany

What about the availability of open source drivers through micromanager? Although it is somewhat recognized that the micromanager API/architecture is not ideal, it is certainly recognized that the large number of already existing drivers is a key strength. How is the availability of drivers for Cockpit?

 How does Cockpit compare to Pycro-manager (Pycro-Manager: open-source software for customized and reproducible microscope control, Pinkard *et al.*, 2021, Nature Methods), ^1^ which enjoys the benefits of the existing micromanager ecosystem while also providing a Python interface?

 Overall, Cockpit seems like a strong contribution. I see a lot of potential, but it is not clear that it is quite competitive with micromanager yet (which is fine, that will come with time). Integration with a tool like napari would be a great choice for broadening the usability of Cockpit. It is nice to see that Cockpit is being tested on multiple systems, although as mentioned it would be good to see a fair comparison between Cockpit's current (and future planned) drivers relative to a mature project like micromanager.

Are the conclusions about the tool and its performance adequately supported by the findings presented in the article?

Yes

Is the rationale for developing the new software tool clearly explained?

Yes

Is the description of the software tool technically sound?

Yes

Are sufficient details of the code, methods and analysis (if applicable) provided to allow replication of the software development and its use by others?

Yes

Is sufficient information provided to allow interpretation of the expected output datasets and any results generated using the tool?

Yes

Reviewer Expertise:

Image analysis, Computer science, Software engineering, Computational biology, Python, Java, C++, Lisp

I confirm that I have read this submission and believe that I have an appropriate level of expertise to confirm that it is of an acceptable scientific standard.

## References

[1] : Pycro-Manager: open-source software for customized and reproducible microscope control. *Nature Methods* .2021;18(3) : 10.1038/s41592-021-01087-6 226-228 10.1038/s41592-021-01087-6 PMC853217633674797

# Response

**Published:** 2021-12-14

**Authors:** Ian Dobbie

**Affiliations:** University of Oxford, UK

Response to review from Authors. 

 What about the availability of open source drivers through micromanager? Although it is somewhat recognized that the micromanager API/architecture is not ideal, it is certainly recognized that the large number of already existing drivers is a key strength. How is the availability of drivers for Cockpit?

*This is an interesting idea and we have considered it. There are two major arguments against this approach. Firstly this would require importing the whole of the uManager infrastructure, the java JVM etc… Secondly our brief exploration appeared to show that matching devices to the fixed device type specific API in Python-Microscope, the library we use to interface directly to the hardware, would involve substantial effort for each device. We felt it was more productive o apply this effort to implementing the device directly in Python. This probably took roughly the same effort and enabled us to avoid the uManager overhead.*

 How does Cockpit compare to Pycro-manager (Pycro-Manager: open-source software for customized and reproducible microscope control, Pinkard *et al.*, 2021, Nature Methods), which enjoys the benefits of the existing micromanager ecosystem while also providing a Python interface?

*We thank the reviewer for pointing out this paper which was published since our original submission and we have now added discussion about Pycro-Manager to the paper. We have revised the manuscript by adding the following text to the discussion section:*

**It should be noted that Pycro-Manager [pinkard2021] has recently been developed and released to allow much closer integration between Python and uManager. This seems likely to fill a similar position as Microscope-Cockpit, allowing direct Python based control of imaging experiments, integration with online analysis and a range of other functionality. As this is a recent development we have not explored this package in any detail.**
